# Successful heart transplantation in an infant with phosphoglucomutase 1 deficiency (PGM1‐CDG)

**DOI:** 10.1002/jmd2.12350

**Published:** 2022-11-22

**Authors:** Ruqaiah Altassan, Dimpna C. Albert‐Brotons, Mohammad Alowain, Zohair Al‐Halees, Jaak Jaeken, Eva Morava

**Affiliations:** ^1^ Department of Medical Genomics, Centre for Genomic Medicine King Faisal Specialist Hospital and Research Centre Riyadh Saudi Arabia; ^2^ College of Medicine Alfasial University Riyadh Saudi Arabia; ^3^ Department of Clinical Genomics Mayo Clinic Rochester Minnesota USA; ^4^ Department of Cardiology, Heart Centre King Faisal Specialist Hospital and Research Centre Riyadh Saudi Arabia; ^5^ Department of Pediatrics University Hospitals Leuven Leuven Belgium

**Keywords:** congenital disorders of glycosylation, D‐galactose, heart transplantation, PGM1‐CDG, ventricular non‐compaction

## Abstract

We report successful heart transplantation in a phosphoglucomutase 1 deficient (PGM1‐CDG) patient. She presented with facial dysmorphism, bifid uvula and structural heart defects. Newborn screening was positive for classic galactosemia. The patient was on a galactose‐free diet for 8 months. Eventually, whole exome sequencing excluded the galactosemia and revealed PGM1‐CDG. Oral D‐galactose therapy was started. Rapid deterioration of the progressive dilated cardiomyopathy prompted heart transplantation at the age of 12 months. Cardiac function was stable in the first 18 months of follow‐up, and hematologic, hepatic, and endocrine laboratory findings improved during D‐galactose therapy. The latter therapy improves several systemic symptoms and biochemical abnormalities in PGM1‐CDG but does not correct the heart failure related to cardiomyopathy. Heart transplantation has so far only been described in DOLK‐CDG.

**Abbreviations**
CDG: congenital disorder(s) of glycosylation
CK: creatine kinase
ECMO: extracorporeal membrane oxygenation
ES: exome sequencing
G‐6‐P: glucose‐6‐phosphate
GALT: galactose‐1‐phosphate uridyl transferase
G‐l‐P: glucose‐1‐phosphate
HPLC: high‐performance liquid chromatography
IGFBP‐3: insulin‐like growth factor binding protein 3
LC–MS/MS: liquid chromatography with tandem mass spectrometry
LVEF: left ventricular ejection fraction
MLPA: multiplex‐ligation dependent probe amplification
PGM1: phosphoglucomutase 1
TBG: thyroid‐binding globulin
TIEF: transferrin isoelectric focusing
ZASP: Z‐band alternatively spliced PDZ motif protein

**Synopsis**

This is the first successful cardiac transplantation in PGM1‐CDG.

## 1 INTRODUCTION

Phosphoglucomutase 1 (PGM1) catalyzes the interconversion of glucose‐1‐phosphate (G‐l‐P) and glucose‐6‐phosphate (G‐6‐P), a reaction at the intersection of glycogenesis, glycolysis and glycosylation.^1^ PGM1‐CDG (OMIM 614921) is an autosomal recessive disorder characterized by congenital defects (cleft lip/palate and bifid uvula), hepatopathy, hypoglycemia, dilated cardiomyopathy, and hypogonadotropic hypogonadism. Myopathy and exercise intolerance are more evident in older age and may be associated with rhabdomyolysis.^2^

Isoelectrofocusing of serum transferrin in PGM1‐CDG usually shows a mixed type 1/2 pattern.^2^

Oral D‐galactose supplementation corrects the defective galactosylation and hypoglycosylation in vitro and in vivo and improves growth, hypoglycemic control, and glycosylation abnormalities.^3^, ^4^, ^5^ Here, we report on a very young PGM1‐CDG patient with dilated cardiomyopathy whose systemic symptoms responded to D‐galactose therapy. However, heart function failed and heart transplantation was successfully performed at the age of 12 months.

## 2 PATIENT REPORT

This 30‐month‐old female is the first child of young consanguineous parents. She was born at 37 weeks of gestation via normal vaginal delivery with a birth weight of 2.9 kg (10th centile). After birth, transthoracic echocardiography, performed because of a systolic murmur, showed congenital pulmonary valve stenosis and a dysplastic pulmonary valve. Her newborn screen was positive for a low galactose‐1‐phosphate uridyl transferase (GALT) activity for which she received a galactose‐restricted diet. She was first seen by us at the age of 6 weeks. Facial dysmorphism consisted of bilateral ptosis, low‐set and posteriorly rotated ears, low posterior hairline, long philtrum, and bifid uvula. There was also a systolic cardiac murmur and mild hepatomegaly. Weight was 4.8 kg (50th centile), length 57.5 cm (75th centile), and head circumference 39.8 cm (75th centile). At the age of 3 months, she underwent a successful balloon valvuloplasty for pulmonary valve stenosis. At the age of 6 months, she was presented to our hospital with heart failure and hypoglycemia (1.6 mmol/L). Echocardiography demonstrated severe congenital mitral valve regurgitation with dilated impaired systolic left ventricular function and non‐compaction cardiomyopathy (Table 1). She was started on anti‐failure medications (captopril and diuretics). She continued to be symptomatic mainly with respiratory distress secondary to the lung congestion, and her left ventricular ejection fraction (LVEF) decreased from 72% to 54% within 15 days of her hospitalization. A mitral valve repair with a mechanical valve was performed but failed to improve her cardiac function. She developed cardio‐pulmonary arrests due to the poor ventricular function and low cardiac output associated with a complete heart block and she required extracorporeal membrane oxygenation (ECMO) support for 4 days. After ECMO removal, her cardiac function remained low (LVEF 10%) and she required continued inotropic support, in addition to an epicardial pacemaker for complete atrioventricular block. During her hospitalization, she had frequent hypoglycemic episodes which required continuous nasogastric (NG) tube feeding.

**TABLE 1:** Summary of clinical, biochemical, and echocardiographic data in a PGM1‐CDG patient before and on galactose supplementation, and before and after heart transplantation

Age	6 weeks	6 months	11 months	12 months	18 months	30 months
Values						
D‐galactose therapy	No	No	No	Yes	Yes	Yes
				*+Heart transplantation*		
NPCRS	23 (moderate)			3 (mild)		
Growth						
Weight kg/SD	4.8	5.8	6.2	8	9.35	12
	0.43	−2.64	−3.90	−2.10	−1.29	−0.95
Length/height cm/SD	57.5	57.50	63	68.20	70	84
	0.9	−3.70	−3.41	−2.41	−2.46	−1.3
Glucose (3.9–6.9 mmol/L)	–	1.9	3.3	5.3	5.9	4.8
TSH (0.27–4.2 mU/L)	–	10.5	6.2	4.1	0.9	2
Coagulation						
INR (0.8–1.1)	–	2.4	4.3	1.1	1.1	1
PT (10.8–13.4 s)	–	31.4	54.9	14.3	15.4	13.7
PTT (30.5–40.4 s)	–	57.4	57.7	38	38.7	42.9
Anti‐thrombin (80–120%)	–	9	68	100	100	–
Protein C	–	NL	NL	NL	NL	–
Protein S	–	NL	NL	NL	NL	–
Serum transaminases						
ALT (10–45 U/L)	57	73	84.6	37.5	10.3	26.6
AST (10–45 U/L)	111	146	383.2	36.6	20.9	32.7
Creatine kinase	NL	NL	NL	NL	NL	NL
Lipid profile	–	NL	NL	NL	NL	NL
Copper	NL	–	–	–	–	–
Ceruloplasmin	NL	–	–	–	–	–
GALT, DBS (3.5 U/g Hb)	2.4	–	2.3	–	2	–
Serum transferrin isoform analysis by HPLC						
Disialotransferrin (<1.7)	NA	NA	5	1.2	2.6	–
Echocardiography	Pulmonary valve stenosis	S/P pulmonary valve balloon dilatation – Severe mitral valve regurgitation – Severely dilated left atrium, – Dilated and hypertrabeculated left ventricle – Non‐compaction changes	S/P mechanical mitral valve replacement – Mild mitral valve regurgitation – Severely dilated left ventricle – Non‐compaction changes	NL	NL	NL
LVEF	72%	54%	10%	72%	NL	NL

Abbreviations: ALT, alanine aminotransferase; AST, aspartate transaminase; DBS, dried blood spot; GALT, galactosyltransferase; INR, international normalized ratio; LVEF, left ventricular ejection fraction; NA, not available; NL, normal; NPCRS, Nijmegen Paediatric CDG Rating Scale; PT, prothrombin time; PTT, partial thromboplastin time; S/P, status post; TSH, thyroid stimulating hormone.

Her growth velocity significantly decreased after the age of 6 months, and she failed to maintain a normal weight over the last 6 months of her first year. Her developmental milestones were affected during the first year of life and she had delay mainly in her motor function and speech. Detailed evaluation by Nijmegen Paediatric CDG Rating Scale (NPCRS)^6^ at the age of 11 month revealed moderate category. Clinical exome sequencing (ES) was carried out and identified a previously reported homozygous missense variant in the PGM1 gene (NM_002633.3: c.1544G>A (p. Arg515Gln)).^6^ With the supportive clinical phenotype and the positive biochemical finding of abnormal CDT% (Table 1), the variant was upgrade to a likely pathogenic. Furthermore, this variant was found in a homozygous status in two different patients from unelated families presented with PGM‐CDG phenotype, the variant's frequency in gnomAD was 0.00001194. Similarly, the frequency in our local database was very low, it was only detected in heterozygous status in five individuals. Parental carrier testing confirmed in trans inheritance. Sequencing and multiplex‐ligation dependent probe amplification (MLPA) for the GALT, GALK1, and GALE genes did not reveal any pathogenic changes.

Biochemical results are shown in Table 1. Abnormal results included increased serum transaminases, thyroid stimulating hormone and disialotransferrin isoform percentage, decreased antithrombin, and prolonged coagulation profile. Beutler test on dried blood spots was repeatedly mildly decreased for GALT activity. This low GALT activity in blood was confirmed by liquid chromatography–tandem mass spectrometry analysis (LC–MS/MS) (21 nmol/h/mg/Hb; reference range: ≥24.5 nmol/h/mg/Hb).

Oral D‐galactose supplementation (0.5 g/kg/day) was started at the age of 11 months. Within a few days, the patient showed significant improvement in her glycemia control, and she was able to be weaned from the continuous NG feeding. Her coagulation profiles, serum transaminase levels, antithrombin, and thyroid function all normalized within a month from starting the therapy and the disialotransferrin percentage decreased.

However, her cardiac function remained unchanged. Inotropic support did not improve the cardiac function and at the age of 12 months, she underwent ABO‐incompatible heart transplantation (donor blood type B, and recipient blood type O). Follow‐up over 18 months post‐transplantation showed markedly improved growth parameters, and overall developmental milestones, NPCRS down to the mild category and heart function (LVEF) normalized. There were no episodes of graft rejection and the immunosuppression medication showed no side effects. In addition, the increment in the D‐galactose dose to 1.5 g/kg/day four times was well tolerated.

## 3 DISCUSSION

We report on a severe, rapidly progressing cardiomyopathy, requiring heart transplantation in a toddler with PGM1‐CDG. She was restricted on her galactose intake for the first half year of life, and only properly diagnosed at the age of 8 months and started on D‐galactose therapy. The initial newborn screen misled the patient's diagnosis and her dietary management. Classic galactosemia usually manifests with feeding problems, failure to thrive, hepatocellular damage, bleeding, and sepsis if untreated.^7^ Apart from the hypoglycemia, our patient initially did not present with the classical presentation of galactosemia. Instead, she had a complex phenotype that led to considering different etiologies and necessitated more comprehensive molecular testing i.e. ES. This indicates the needs for clinicians to broaden the differential diagnosis of the abnormal GALT screening in the presence of the unusual congenital anomalies. The failure in her weight spurt was evident after her first symptomatic cardiac presentation at the age of 6 months. Her serum transaminases and coagulation profiles were mildly elevated at the age of 6 weeks but then progressively increased over her first year of life. In retrospect, the galactose restricted diet could have contributed to the worsening laboratory results, her frequent hypoglycemic episodes, and her growth regression.

The standard GALT activity assay by Beutler spot test involves three enzymatic reactions: phosphoglucomutase, glucose 6‐phosphate dehydrogenase, and 6‐phosphogluconate dehydrogenase.^8^ False‐positive GALT screening results can be seen in glucose‐6‐phosphate dehydrogenase deficiency, citrin deficiency, Fanconi–Bickel disease, and liver disease.^9^, ^10^ Low PGM1 activity can also decrease the GALT activity level as was seen by the standard Beutler test in eight PGM1‐CDG patients; corrected levels were observed in the same patients' samples after adding PGM1 from an animal's heart.^11^

Cardiac involvement is not uncommon in CDG but the exact role of defected glycosylation in the heart structure and function is not yet defined.^12^ In PGM1‐CDG specifically, cardiac manifestation accounted for 50% of patients, it can be structural, functional, and or conductive heart defects with risk of evolvement over the patients' lifetime.^11^, ^13^, ^14^, ^15^, ^16^, ^17^ Dilated cardiomyopathy is the most common reported cardiac manifestation with variable age of onset, severity, and progression, this followed by restrictive cardiomyopathy.^2^, ^18^ Risk of sudden death with cardiomyopathy has been stated in PGM1‐CDG.^16^ Our patient manifested with both structural congenital anomalies and progressive functional defects (dilated cardiomyopathy). The pathophysiology of the cardiac involvement in PGM1 deficiency is not well understood. It is known that N‐glycosylation plays an important role in mediating many cardiac transmembrane interactions.^19^ For heart functionality, the Z‐disc in the cardiac sarcomeres is rich in proteins important for maintaining the microfilament structure and the mechanical connections including the Z‐band alternatively spliced PDZ motif protein/(ZASP)/Cypher.^20^ In conjunction, PMG1 is very abundant and highly expressed in the heart tissue which is explained by the high need for energy production for the heart function. Binding of PGM1 to ZASP/Cypher is essential under stress. Impaired ZASP/Cypher‐PGM1 binding has been linked to dilated cardiomyopathy and heart failure.^11^ PGM1 deficiency is also associated with abnormal glycogen metabolism, which could also affect cardiac function.^21^

The lack of genotype–phenotype correlation in PGM1‐CDG is postulated to be due to the existence of other PGM isoforms.^2^ The detected homozygous variant in our patient was previously reported in three relatives; two siblings and their cousin who presented mainly with facial dysmorphism, hypoglycemia, high liver transaminases, and high creatine kinase; none presented with cardiac abnormalities.^22^

Most of the cardiomyopathies in CDG are managed medically and the outcome is variable among affected patients. Status quo, progression as well as regression of cardiac function have been reported in PGM1‐CDG.^2^, ^18^ There is limited data on the particular effect of the D‐galactose therapy on the heart function. In one patient with restrictive cardiomyopathy, no changes were observed on his heart while he was on D‐galactose supplement.^18^ No PGM1‐CDG patient has yet received heart transplantation but it was considered in four patients presented with cardiomyopathy.^11^ Successful heart transplantation was reported in four patients with severe dilated cardiomyopathy due to DOLK‐CDG.^23^, ^24^

In conclusion, heart transplantation was a lifesaving decision for our patient who was, fortunately, able to receive the new heart at the age of 1 year. In addition, D‐galactose played a major role in controlling her other systemic phenotypes. with multiple congenital anomalies who screen positive for galactosemia there is need to consider other diagnoses.

## FUNDING INFORMATION

Eva Morava is supported by the grant titled Frontiers in Congenital Disorders of Glycosylation (1U54NS115198‐01) from the National Institute of Neurological Diseases and Stroke (NINDS) and the National Center for Advancing Translational Sciences (NCATS), National Institute of Child Health and Human Development and the Rare Disorders Clinical Research Network (RDCRN), at the National Institute of Health.

## CONFLICT OF INTEREST

Ruqaiah Altassan, Dimpna C. Albert‐Brotons, Mohammad Alowain, Zohair Al‐Halees, Jaak Jaeken, and Eva Morava have approved the manuscript and declare no conflict of interest. They did not receive reimbursements/fees/funds/salaries from an organization that may in any way gain or lose financially from the results reported in the reviewed manuscript in the last 5 years and have no other competing financial or non‐financial interests, as outlined in the JIMD Conflict of Interest form.

## ETHICS STATEMENT

All procedures followed were in accordance with the ethical standards of the responsible committee on human experimentation (institutional and national) and with the Helsinki Declaration of 1975, as revised in 2000. Written informed consent was obtained from the patient's parent for collection of samples and publication of medical data.

## Data Availability

This manuscript has no associated data.
